# Genome Sequences of Allochromatium palmeri and Allochromatium humboldtianum Expand the *Allochromatium* Family Tree of Purple Sulfur Photosynthetic Bacteria within the *Gammaproteobacteria* and Further Refine the Genus

**DOI:** 10.1128/MRA.00774-20

**Published:** 2020-08-13

**Authors:** John A. Kyndt, Terry E. Meyer

**Affiliations:** a College of Science and Technology, Bellevue University, Bellevue, Nebraska, USA; b Department of Chemistry and Biochemistry, The University of Arizona, Tucson, Arizona, USA; Georgia Institute of Technology

## Abstract

New genomes of two *Allochromatium* strains were sequenced. Whole-genome and average nucleotide identity based on BLAST (ANIb) comparisons show that Allochromatium humboldtianum is the nearest relative of Allochromatium vinosum (ANIb, 91.5%), while both Allochromatium palmeri and Thermochromatium tepidum are more distantly related (ANIb, <87%). These new sequences firmly establish the position of *Allochromatium* on the family tree.

## ANNOUNCEMENT

Chromatium vinosum (now *Allochromatium vinosum*) is the prototypic purple sulfur bacterium, and it is the only species in the genus to have had a genome sequence determined (1). Moreover, there are several genera that are fairly closely related to *Allochromatium*, including *Thiocystis*, *Thermochromatium*, *Chromatium*, and *Thiorhodococcus* (2), although the relationships are not clear despite single-gene comparisons (3); therefore, a whole-genome comparison including multiple *Allochromatium* species is needed.

Allochromatium palmeri DSM 15591^T^ was originally isolated from a cave system in the Bahamas (4), while Allochromatium humboldtianum DSM 21881^T^ was isolated from marine sediments in Peru (5). Cultures were grown and genomic DNA was prepared by the Deutsche Sammlung von Mikroorganismen und Zellkulturen (DSMZ). DNA analysis showed *A*_260_/*A*_280_ ratios of 1.60 for A. palmeri and 1.96 for A. humboldtianum. The sequencing libraries were prepared using the Illumina Nextera DNA Flex library preparation kit and were sequenced by an Illumina MiniSeq sequencer using 500 μl of a 1.8 pM library. Paired-end (2 × 150-bp) sequencing generated 2,433,982 reads and 192 Mbp for A. palmeri and 3,349,346 reads and 252.2 Mbp for A. humboldtianum. Quality control of the reads was performed using FastQC within BaseSpace (version 1.0.0; Illumina), using a k-mer size of 5 and contamination filtering. We assembled the genome *de novo* through PATRIC (6) using SPAdes (version 3.10.0) (7) for A. palmeri and Unicycler for A. humboldtianum. The assembly yielded 196 contigs (>300 bp) and an *N*_50_ value of 74,142 bp for A. palmeri (45× coverage), while A. humboldtianum was assembled into 86 contigs with an *N*_50_ value of 305,111 bp (55× coverage). The A. palmeri genome had a GC content of 62.5% and a length of 4,272,782 bp, whereas the A. humboldtianum genome had a GC content of 63.9% and a length of 4,584,820 bp. The genomes were annotated using the RAST tool kit (RASTtk) (8) within PATRIC (6). This annotation showed A. palmeri to have 4,134 coding sequences and 45 tRNAs and A. humboldtianum to contain 4,391 coding sequences and 47 tRNAs. Default parameters were used for all software applications unless otherwise noted.

A JSpeciesWS comparison (9) of average nucleotide identity based on BLAST (ANIb) showed 86.8% identity between A. palmeri and A. humboldtianum (Table 1). A. humboldtianum is closer to Allochromatium vinosum with 91.5% ANIb, while A. palmeri showed 86.6% ANIb. All of these ANIb values are clearly below the proposed 95% cutoff value for genome definition of a species (9). Thermochromatium tepidum is about equidistant from all three of the *Allochromatium* species; however, Allochromatium warmingii appears to be more distant from all of them.

**TABLE 1:** ANIb comparisons

Strain	ANIb (%) with strain:			
	*A. vinosum* DSM 180^T^	A. humboldtianum DSM 21881^T^	A. palmeri DSM 15591^T^	*T. tepidum* ATCC 43061^T^
A. humboldtianum DSM 21881^T^	91.5			
A. palmeri DSM 15591^T^	86.6	86.8		
*T. tepidum* ATCC 43061^T^	84.3	84.9	82.2	
*A. warmingii* DSM 173^T^	76.6	76.5	76.4	74.7

**FIG 1:**
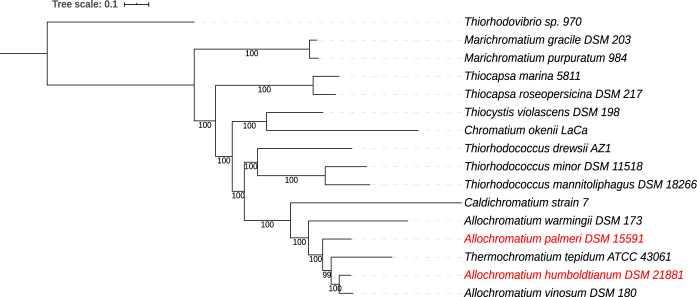
Whole-genome-based phylogenetic tree of all sequenced *Allochromatium* and related species. The phylogenetic tree was generated using the Codon Tree method within PATRIC (6), which used PATRIC global protein families (PGFams) as homology groups; 467 PGFams were found among these selected genomes using the Codon Tree analysis, and the aligned proteins and coding DNA from single-copy genes were used for RAxML analysis (10, 11). The support values for the phylogenetic tree are shown on the tree branches and were generated using 100 rounds of the rapid bootstrapping option of RaxML. *Thiorhodovibrio* was used as an outgroup. Interactive Tree Of Life (iTOL) was used for the tree visualization (17).

Whole-genome-based phylogenetic analysis was performed with RAxML within PATRIC (10, 11) using all of the *Allochromatium* and related genomes (1, 12–16). This analysis grouped all of the *Allochromatium* species (Fig. 1); however, it also placed Thermochromatium tepidum within this group. Consistent with the ANIb analysis, A. warmingii is more distant from the other *Allochromatium* species. Further genetic and physiological studies may be needed to determine whether a nomenclature change of the latter species is warranted. The addition of these new *Allochromatium* genomes has substantially strengthened the phylogenetic tree of this genus.

### Data availability.

These whole-genome shotgun projects have been deposited in DDBJ/ENA/GenBank under the accession numbers WNKT00000000 for Allochromatium palmeri and JABZEO000000000 for Allochromatium humboldtianum. The versions described in this paper are versions WNKT010000000 and JABZEO010000000. The raw sequencing reads have been submitted to SRA, and the accession numbers are SRR12110462 for Allochromatium palmeri and SRR12110432 for Allochromatium humboldtianum.
